# Protective and ameliorative effect of sea buckthorn leaf extract supplementation on lead induced hemato-biochemical alterations in Wistar rats

**DOI:** 10.14202/vetworld.2016.929-934

**Published:** 2016-09-02

**Authors:** Rizwana Zargar, Pratiksha Raghuwanshi, Ankur Rastogi, Aditi Lal Koul, Pallavi Khajuria, Aafreen Wahid Ganai, Sumeet Kour

**Affiliations:** 1 Division of Veterinary Physiology and Biochemistry, Sher-e-Kashmir University of Agricultural Sciences and Technology of Jammu, RS Pura - 181 102, Jammu and Kashmir, India; 2 Division of Animal Nutrition, Sher-e-Kashmir University of Agricultural Sciences and Technology of Jammu, RS Pura - 181 102, Jammu and Kashmir, India; 3 Division of Veterinary Public Health and Epidemiology, Sher-e-Kashmir University of Agricultural Sciences and Technology of Jammu, RS Pura - 181 102, Jammu and Kashmir, India

**Keywords:** hemato-biochemical, lead, sea buckthorn leaf extract, Wistar rats

## Abstract

**Aim:**

To evaluate the protective and ameliorative effect of aqueous sea buckthorn leaf extract (SLE) on hemato-biochemical profile in lead intoxicated Wistar rats.

**Materials and Methods:**

An experiment was conducted for 60 days. 36 adult male Wistar rats with a mean body weight of 177.8±12.6 g were divided into five groups and were subjected to various daily oral treatment regimens. Group I served as a negative control receiving only feed and water, Group II (positive control for lead) received lead acetate at 250 ppm in drinking water, and Group III (positive control for SLE) received SLE at 100 mg/kg b.wt. Animals in Group IV received a combination of lead acetate at 250 ppm in drinking water for the first 45 days and SLE at 100 mg/kg b.wt. throughout the experimental period of 60-day, and in Group V for the last 15 days of the trial after the administration of lead acetate until the first 45 days of the trial to study the protective and ameliorating effects of SLE, respectively. Blood samples were collected from retro-orbital fossa of each rat on 0^th^, 45^th^, and 60^th^ day of the experiment for hemato-biochemical analysis including hemoglobin (Hb), packed cell volume (PCV), serum total protein, albumin, globulin, albumin:globulin ratio, cholesterol, urea, and creatinine.

**Results:**

Significantly (p<0.01) lower levels of serum total proteins and albumin, and a significantly (p<0.01) higher serum cholesterol, urea and creatinine levels were observed in Group II (lead intoxicated group) in comparison to Group I (negative control). Administration of SLE at 100 mg/kg body wt. to lead intoxicated Wistar rats resulted in normalization of almost all the biochemical parameters studied in both the treatment Groups, i.e., IV and V (protective and ameliorative). However, the effects were more pronounced in the protective group. No effects of SLE supplementation were observed on Hb levels. PCV levels improved in protective groups, but no effect was observed in ameliorative group in comparison to lead intoxicated groups.

**Conclusion:**

SLE administration at 100 mg/kg b.wt. to lead intoxicated Wistar rats may be used to protect/ameliorate lead induced biochemical alterations in Wistar rats.

## Introduction

Lead is one of the most common environmental pollutants [1] and has continued to cause health hazards in animals and humans in many parts of the world [2]. The exposure to lead can occur from a multitude of sources such as soil, air, water, and industrial pollutants. Either agricultural or industrial production or accidental or deliberate misuse has resulted in the excessive amount of lead in animal feed and feedstuffs [3,4]. In recent years, lead has become a regulatory concern among pharmacologists, environmental scientists, and clinicians due to its continuous emission from industrial sources and automobile exhausts and its pharmacological behavior to remain bound to mammalian tissues [5]. It has many undesired effects including neurological [6,7], behavioral [8,9], immunological [10-13], renal [14-16], hepatic [15], and especially hematological dysfunctions [17,18].

Oxidative damage to various organs including heart, liver, kidneys, reproductive organs, brain, and erythrocytes has been reported to be the basal cause of lead mediated damage [19]. Lead causes oxidative stress by inducing the generation of reactive oxygen species (ROS) and weakening the antioxidant defense system of cells [20]. ROS are highly reactive to membrane lipids, protein, and DNA and are believed to be the major contributing factors causing stress injuries leading to rapid cellular damage [21,22] including peroxidation damage to erythrocyte membranes [23].

Herbal medicines derived from plant extracts are being increasingly used in treating a wide variety of metal toxicities [24]. Aqueous sea buckthorn (SBT) leave extract (SLE) has been shown to have protective effect against chromium induced oxidative stress in *in vivo* as well as in *in vitro* studies [25,26]. *Hippophae rhamnoides* L. subspecies turkestanica (family Elaeagnaceae) commonly known as SBT is a branched and thorny nitrogen-fixing deciduous shrub, native to Europe and Asia, particularly in Ladakh division of Jammu and Kashmir, and Himachal Pradesh. In the recent years, diverse pharmacological activities of SBT plant such as antioxidant, cryoprotective, antistress, immunomodulatory, hepatoprotective, radioprotective, antiatherogenic, antitumor, antimicrobial, and tissue regeneration have been reported [27]. The leaves of the plant are rich in several nutrients and bioactive substances, mainly phenolic compounds including catechin, polyphenols, carotenoid lycopene, bioflavonoids, and coumarins [28-30]. Keeping the above in view, an attempt was made to access the protective and ameliorative effect of SLE on lead induced toxicity.

## Materials and Methods

### Ethical approval

All rats were maintained under standard environmental conditions with *ad libitum* feed and water. The animals were treated humanely during the whole period of the experimental study, and the work was approved by the Institutional Animal Ethics Committee vide No. 862/AC/04/CPCSEA on ethical standards in animal experimentation.

The Wistar rats weighing between 150 and 200 g used in this study were procured from Indian Institute of Integrative Medicine, Council of Scientific and Industrial Research Lab, Jammu, India.

### Chemicals

Lead acetate (99.9% pure) was purchased from Hi-Media Labs Mumbai. All other chemicals used in the study were of extra pure quality and purchased from Hi-media, S.D. Fine Chem. Pvt. Ltd., Qualigens Chem. (Mumbai, India), and E. Merck (Mumbai, India).

### Collection and preparation of aqueous SLE

SBT leaves were collected from the Ladakh division of Jammu and Kashmir in the month of August. The collected leaves were duly verified by the subject expert from University of Jammu, Jammu. After shade drying, leaves were pulverized to powder form, and SLE was prepared by cold percolation method [31]. Dried leaf powder was soaked in distilled water (1:5 w/v) at room temperature (25°C). After 24 h, the supernatant was collected and the residue was resoaked in fresh water. The process was repeated four times for complete extraction. The supernatants were pooled, filtered through muslin cloth and filtrate was centrifuged at 8000 g at 4°C. After centrifugation, the supernatant obtained was concentrated in a rotary evaporator to obtain a semisolid consistency of SLE and stored at −20°C for further use.

### Experimental design

The experimental rats were randomly divided into five groups of eight rats in Group I and seven rats in remaining four groups and were subjected to various daily treatment regimens for 60 days. Group I (negative control) received no treatment except normal feed and water, Group II received lead acetate at 250 ppm in drinking water, Group III received SLE at 100 mg/kg body weight orally, and Group IV received combination of lead acetate at 250 ppm for the first 45 days and SLE at 100 mg/kg body weight throughout the experimental period and to Group V for the last 15 days of the trial after the administration of lead acetate until the 45^th^ day of the trial to study the protective and ameliorating effect of SLE, respectively. The administration of the toxicant was carried out between 9:30 and 10:30 am daily. All the rats were weighed at weekly intervals during exposure with toxicants and necessary corrections in dosages were made accordingly. Blood samples were collected from retro-orbital fossa of each rat on 0^th^, 45^th^, and 60^th^ day of the experiment for hemato-biochemical analysis. Serum was separated and stored in deep freeze (−20°C) for further biochemical analysis.

### Hemato-biochemical analysis

Hemoglobin (Hb) and packed cell volume (PCV) analysis were performed as per cyanomethemoglobin method [32] and microhematocrit method [33], respectively. Biochemical analysis for total protein, albumin cholesterol, urea, and creatinine was performed according to manufacturer’s instructions using Agappe Diagnostic Pvt. Ltd. kits.

### Statistical analysis

Statistical analysis was performed using generalized linear model analysis of variance [34] and Duncan’s multiple range test [35].

## Results and Discussion

Hematological parameters, *viz*., Hb and PCV are presented in Table-1, and biochemical parameters, *viz*., cholesterol, urea, creatinine, total protein, and albumin are presented in Table-2.

**Table-1:** Protective and ameliorative effects of SLE supplementation on hematological parameters in lead intoxicated rats.

Group	0^th^ day	45^th^ day	60^th^ day	Group Mean±SEM
Hb (g/dl)				
I	13.22±0.61	13.58±0.47	13.04±0.43	13.26^b^±0.28
II	13.22±0.61	10.63±0.30	9.33±0.32	10.97^a^±0.45
III	13.22±0.61	13.38±0.30	13.26±0.27	13.28^b^±0.22
IV	13.22±0.61	10.35±0.24	11.47±0.37	11.67^a^±0.36
V	13.22±0.61	10.07±0.18	10.23±0.19	11.12^a^±0.39
PCV (%)				
I	38.50±1.18	40.12±1.43	38.65±1.28	39.05^c^±0.72
II	38.50±1.18	31.13±0.82	27.47±1.01	32.11^a^±1.22
III	38.50±1.18	39.25±1.11	39.16±0.73	38.98^c^±0.55
IV	38.50±1.18	30.50±0.73	33.80±1.13	34.24^b^±0.95
V	38.50±1.18	31.42±1.15	30.25±0.50	33.22^ab^±1.00

Figures bearing different superscripts differ significantly (p<0.05). Hb: Hemoglobin, PCV: Packed cell volume, SEM: Standard error of mean, SLE: Sea buckthorn leaf extract

**Table-2:** Protective and ameliorative effects of SLE supplementation on biochemical parameters in lead intoxicated rats.

Group	0^th^ day	45^th^ day	60^th^ day	Group Mean±SEM
TP (g/dl)				
I	5.66±0.05	5.91±0.16	6.05±0.10	5.89^d^±0.07
II	5.66±0.05	5.11±0.23	4.20±0.13	4.95^a^±0.17
III	5.66±0.05	5.64±0.23	6.08±0.08	5.81^d^±0.09
IV	5.66±0.05	5.27±0.08	5.71±0.17	5.56^c^±0.08
V	5.66±0.05	5.06±0.08	4.88±0.10	5.18^b^±0.09
Albumin (g/dl)				
I	4.27±0.05	4.32±0.19	4.00±0.14	4.18^dc^±0.08
II	4.27±0.05	3.16±0.10	3.22±0.13	3.53^a^±0.13
III	4.27±0.05	4.14±0.10	4.37±0.15	4.27^d^±0.07
IV	4.27±0.05	3.61±0.16	4.24±0.08	4.05^c^±0.09
V	4.27±0.05	3.23±0.14	3.73±0.07	3.74^b^±0.11
Globulin (g/dl)				
I	1.39±0.06	1.59±0.10	2.05±0.14	1.71±0.09
II	1.39±0.06	1.77±0.24	0.98±0.15	1.36±0.12
III	1.39±0.06	1.50±0.26	1.71±0.19	1.54±0.11
IV	1.39±0.06	1.66±0.12	1.65±0.15	1.57±0.07
V	1.39±0.06	1.83±0.15	1.15±0.08	1.44±0.09
A:G				
I	3.10±0.15	2.79±0.25	2.04±0.20	2.59±0.16
II	3.10±0.15	2.05±0.41	4.16±1.11	3.16±0.46
III	3.10±0.15	4.33±1.91	2.85±0.44	3.40±0.61
IV	3.10±0.15	3.24±0.22	2.76±0.39	2.70±0.18
V	3.10±0.15	1.84±0.21	3.37±0.27	2.80±0.20
Urea (mg/dl)				
I	15.21±1.57	16.14±1.66	16.52±0.85	16.01^a^±0.73
II	15.21±1.57	31.65±1.18	36.88±1.03	28.38^d^±2.28
III	15.21±1.57	16.14±1.33	16.08±1.04	15.82^a^±0.72
IV	15.21±1.57	22.65±4.32	20.97±0.81	19.68^b^±1.57
V	15.21±1.57	30.89±0.80	28.81±0.77	25.71^c^±1.71
Creatinine (mg/dl)				
I	0.78±0.10	0.70±0.10	0.58±0.09	0.68^a^±0.06
II	0.78±0.10	1.22±0.03	2.64±0.14	1.61^c^±0.20
III	0.78±0.10	0.65±0.11	0.67±0.07	0.70^a^±0.05
IV	0.78±0.10	0.85±0.02	0.73±0.04	0.79^a^±0.04
V	0.78±0.10	1.08±0.07	0.93±0.04	0.93^b^±0.04
Cholesterol (mg/dl)				
I	65.54±4.66	75.41±3.67	69.15±2.17	69.95^a^±2.07
II	65.54±4.66	100.68±4.14	106.98±3.14	91.91^c^±4.78
III	65.54±4.66	81.04±3.53	66.84±1.58	70.91^a^±2.44
IV	65.54±4.66	92.38±2.38	84.49±0.91	81.00^b^±3.04
V	65.54±4.66	104.12±2.71	99.38±0.92	90.19^c^±4.30

Figures bearing different superscripts differ significantly (p<0.05). A:G: Albumin:globulin, TP: Total protein, SLE: Sea buckthorn leaf extract, SEM: Standard error of mean

Administration of lead acetate resulted in significant (p<0.01) reduction of Hb and PCV values in lead treated Group II as compared to control Group I. This was in agreement with Helmy *et al*. [36] who reported microcytic hypochromic anemia in lead intoxicated rats. Lead causes anemia by impairment of heme synthesis and an increased rate of red blood cell (RBC) destruction [37]. Sharma *et al*. [38] reported interference of lead in heme biosynthesis which is characterized by several enzyme blockades exerting the negative effect on the hematopoietic system. Lead exposure also causes Hb oxidation, leading to RBC hemolysis [39]. Similar findings have been reported by previous workers [40]. In this study, no improvement was observed in Hb levels after the treatment of lead intoxicated rats with SLE at 100 mg/kg b.wt in both protective and ameliorative group. The PCV levels, however, were significantly (p<0.01) improved in protective group when compared to lead treated group, but not in the ameliorative group. Thus, it appears that SLE treatment appears to be ineffective in giving protection as well as amelioration against lead induced depression in Hb levels. However, the protective effects of SLE are very much observed in lead treated rats in terms of PCV levels. These effects might be attributed to the antioxidant properties of SBT leaves [41].

Total protein level is a rough measure of protein status. It also reflects major functional changes in kidney and liver functions. A significant (p<0.01) decrease in serum total proteins and albumin levels in lead-treated animals was observed in this study. Similar observations were reported by other workers [42]. Toxicity or damage to the liver in any form may result in decreased levels of total protein in blood [43]. Deposition of lead leads to the liver injury and consequent disturbances in protein metabolism [44]. Reduced plasma albumin levels in lead intoxicated rats show poor liver function and resultant impaired albumin synthesis [4]. A significant (p<0.01) improvement in total protein levels after SLE treatment in lead intoxicated animals may be due to its hepatoprotective role by normalization of protein synthesis [45] and also due to its antioxidant potential [46]. No significant variations in globulin and albumin:globulin ratio in lead treated rats as well as in both the treatment groups were observed in this study. Similar findings were reported by Ibrahim *et al*. [4]. No changes in globulin and A:G ratio seems to show the general well-being of animals with no exposure to any kind of infection.

Serum creatinine and blood urea nitrogen (BUN) levels are useful marker of a regular filtration in the kidney [47]. Creatinine levels usually rise later than BUN, and thus reflect more chronic condition [46]. In this study, the significant (p<0.01) increase in serum creatinine and urea levels in lead treated group are in agreement with the findings of other studies [48-50]. Metabolism of xenobiotics, such as lead, being an energetic process by utilizing phosphocreatinine for energy generation, and therefore, explain the increased level of creatinine in this study [51]. Compromised kidney will not be able to perform the urea excretion function leads to uremia condition [52]. SLE treatment of lead intoxicated rats resulted in normalization of serum urea and creatinine values in protective as well as ameliorative group. These results may be due to the nephron-protective effects of SLE [45] by normalization of glomerular filtration rate resulting in normal levels of urea and creatinine in SLE treated lead intoxicated rats. These effects of SLE may again also be correlated to antioxidant properties of SBT leaf extract [41].

There was a significant (p<0.01) increase in serum cholesterol in the lead-treated rats in the present study. These findings were in agreement with Abdou and Hassan [53]. It is possible that the cholesterol synthesis and transport pathways may adversely affect in lead treated subjects leading to change in cholesterol level [54]. Supplementation of SLE in lead intoxicated rats resulted in normalization of elevated cholesterol levels. This might be due to the presence of several nutrients and bioactive substances phytochemicals in SBT leaves which mainly include flavonoids, carotenoids, free and esterified sterols, triterpenols, and isoprenoids [41]. The polyunsaturated fatty acid content of SBT may also be responsible for lowering effects of cholesterol. The ability of scavenging free radicals and antioxidant properties of the SLE may also participate in the hypolipidemic activity by inactivating hepatic 3-hydroxy-3-methyl-glutaryl-coenzyme A reductase, a key enzyme, in cholesterol synthesis [41]. All the above beneficial effects of aqueous extract of SBT leaves may be attributed to the potent antioxidant property. SBT leaves of has been claimed to be rich source of various antioxidant substances including flavonoids and other polyphenolic compounds. The main antioxidant substances reported to be present in SBT leaves are flavonoids, quercetin, isorhamnetin, and flavonols such as epicatechin and leucoanthocyanidins [51].

## Conclusion

In the present investigation, we conclude that the supplementation of aqueous extract of SBT leaves at 100 mg/kg b.wt. for 60 days appears to give the effective protection and amelioration against lead induced biochemical alteration in the Wistar rats. Although, the protection was more effective than amelioration.

## Authors’ Contributions

PR and ALK: Designed the research work and provided the technical guidance. RZ: Conducted the research work. PK: Provided necessary help for animal experimentation. AR: Carried out all the statistical analysis. AWG and SK: Helped in laboratory analysis. PR revised the manuscript. All authors read and approved the final manuscript.
